# Epidemiology and transmission characteristics of early COVID-19 cases, 20 January–19 March 2020, in Bavaria, Germany

**DOI:** 10.1017/S0950268821000510

**Published:** 2021-03-02

**Authors:** S. Böhm, T. Woudenberg, D. Chen, D. V. Marosevic, M. M. Böhmer, L. Hansen, J. Wallinga, A. Sing, K. Katz

**Affiliations:** 1 Bavarian Health and Food Safety Authority, Oberschleissheim, Germany; 2 ECDC Fellowship Programme, Field Epidemiology path (EPIET), European Centre for Disease Prevention and Control (ECDC), Stockholm, Sweden; 3 Postgraduate Training for Applied Epidemiology (PAE), Robert Koch Institute, Berlin, Germany; 4 National Institute for Public Health and the Environment (RIVM), Bilthoven, Netherlands; 5 Institute of Social Medicine and Health Systems Research, Otto-von-Guericke-University Magdeburg, Magdeburg, Germany; 6 Department of Biomedical Data Sciences, Leiden University Medical Center, Leiden, Netherlands

**Keywords:** COVID-19, epidemiology, infectious disease epidemiology, outbreaks, pandemic

## Abstract

Severe acute respiratory syndrome-coronavirus-2 (SARS-CoV-2) led to a significant disease burden and disruptions in health systems. We describe the epidemiology and transmission characteristics of early coronavirus disease 2019 (COVID-19) cases in Bavaria, Germany. Cases were reverse transcription polymerase chain reaction (RT-PCR)-confirmed SARS-CoV-2 infections, reported from 20 January−19 March 2020. The incubation period was estimated using travel history and date of symptom onset. To estimate the serial interval, we identified pairs of index and secondary cases. By 19 March, 3546 cases were reported. A large proportion was exposed abroad (38%), causing further local transmission. Median incubation period of 256 cases with exposure abroad was 3.8 days (95%CI: 3.5–4.2). For 95% of infected individuals, symptom onset occurred within 10.3 days (95%CI: 9.1–11.8) after exposure. The median serial interval, using 53 pairs, was 3.5 days (95%CI: 3.0–4.2; mean: 3.9, s.d.: 2.2). Travellers returning to Germany had an important influence on the spread of SARS-CoV-2 infections in Bavaria in early 2020. Especially in times of low incidence, public health agencies should identify holiday destinations, and areas with ongoing local transmission, to monitor potential importation of SARS-CoV-2 infections. Travellers returning from areas with ongoing community transmission should be advised to quarantine to prevent re-introductions of COVID-19.

## Introduction

As of 22 December 2020, 75 129 306 cases of coronavirus disease 2019 (COVID-19) have been reported worldwide, including 561 617 deaths [1]. COVID-19 is caused by the novel severe acute respiratory syndrome-coronavirus-2 (SARS-CoV-2). This virus was identified as the infectious agent of the initial outbreak of viral pneumonia in Wuhan, China, in early January 2020 [2]. In March 2020, coronavirus disease 2019 (COVID-19) was declared a worldwide pandemic [3].

Since late February 2020, most new cases were reported from outside China, with an increasing proportion of these reported from the European Union (EU)/European Economic Area (EEA) countries [4, 5]. The first documented transmission of COVID-19 in Europe occurred in Bavaria in late January 2020, and comprised a cluster of 16 cases [6, 7]. While the last case of this cluster was reported on 11 February, new cases continued to be reported on 27 February and onwards.

Epidemiological parameters, such as the incubation period and the serial interval of SARS-CoV-2, are key factors to characterise the spread of the virus in order to guide interventions, e.g. the duration of quarantine of contact persons, and to quantify the effect of interventions on transmission. Concerted efforts to contain disease spread in the federal state of Bavaria, Germany, included comprehensive contact tracing and testing of contact persons, resulting in extensive data on transmission patterns [6, 8]. As additional information was available on potential exposure intervals and transmission chains for cases affected during the first few weeks of the spread, we characterised the epidemiology and transmission parameters of 3546 cases of COVID-19 reported in Bavaria, Germany, between 20 January and 19 March 2020.

## Methods

### Surveillance

In Germany, notification of SARS-CoV-2 infections was mandatory since 30 January 2020 [9]. A case was defined as a person with a laboratory confirmation by detection of SARS-CoV-2 by reverse transcription polymerase chain reaction (RT-PCR) [10]. Initially in Bavaria, most samples were tested at the Public Health Microbiology (PHM) laboratory of the Bavarian Health and Food Safety Authority (LGL) [6]. As the epidemic advanced, additional private, hospital and university laboratories offered SARS-CoV-2 testing.

For the first 580 cases, the state public health authority maintained a dedicated manual line list to record notifications of SARS-CoV-2 cases in Bavaria, and subsequently switched to using the standard electronic surveillance system. The manual line list included additional information received through event and laboratory reports, as well as contact tracing data from local health authorities. Information on transmission chains between some cases was available.

Testing strategy in the German federal state Bavaria (~13 million inhabitants) was as follows: Individuals who had cumulative face-to-face contact for at least 15 min with a person with a laboratory-confirmed SARS-CoV-2 infection, or direct contact with body fluids of a case, were deemed contacts and were eligible for testing, irrespective of symptoms. Individuals with respiratory symptoms returning from defined risk areas were also eligible for testing [11]. Upon identification of persons eligible for testing, a first upper respiratory sample was taken. Another sample was taken of contact persons who developed symptoms during a 14-day quarantine and irrespective of signs and symptoms, a final sample was collected at the end of the quarantine. From 3 March onwards, the final sample was no longer required, and from 19 March onwards, testing of asymptomatic contact persons was discontinued.

Individuals who reported travel outside of Bavaria, Germany up to 28 days before symptoms onset and were presumably exposed outside of Bavaria, were therefore considered to have been exposed during travels. Until 19 March, the presumed exposure of most reported cases in Bavaria could still be linked to either a stay abroad or contact to a known case, and percentage of reported symptomatic COVID-19 cases ranged between 40% and 80% [12]. Meanwhile, we know community transmission was present in February and early March in some popular travel destinations [13]. Thus, it is most likely that these cases were exposed during their travels outside of Bavaria, Germany, thus, providing a confined window of exposure.

### Descriptive epidemiology

We described reported cases in terms of geographic distribution, as well as symptom status and demographic characteristics. We defined cases as asymptomatic, if clinical information was available, and none of the following symptoms was reported: coughing, diarrhoea, dyspnoea, fever, pneumonia, sore throat and runny nose.

Differences between proportions were tested using either the Chi-squared test or Fisher's exact test. For all analyses, we used the statistical software program R [14]. Visualisations were made using the *ggplot2* package [15]; maps were made using the *tmap* package [16] and networks with the *igraph* package [17].

### Incubation period and serial interval

We estimated the incubation period using cases exposed outside of Bavaria as defined above, who had complete data on both travel period and day of symptom onset. From reported travel histories and symptom onset dates, we inferred the earliest and latest point of exposure, and subsequently the minimum and maximum incubation period of these cases, as has been done previously [18]. To account for long travel periods, and assuming the earliest point of exposure to be on 1 February 2020, we corrected earlier points of exposure to 1 February. For individuals of a family who travelled together, of whom we presume continuous contact after their travels, the last potential point of exposure was set equal to their own day of symptom onset to account for a later point of infection through an infectious family member. We fitted three parametric distributions (gamma, log-normal and Weibull) used to describe incubation periods [18, 19] to the interval data of possible points of exposure. The parametric distribution with the highest log-likelihood was reported and also applied to further analysis using stratified subsets to compare means. We used the *icenReg* package to estimate parameters of the fitted distributions [20]. Confidence intervals were obtained using 1000 parametric bootstrap samples.

To estimate the serial interval, we identified pairs of index and secondary cases from the manual line list, and included those with known dates of symptom onset. We selected only those secondary cases who had a documented exposure to the index case and where no other potential source of infection was known. We fitted the gamma, log-normal and Weibull distribution using maximum likelihood estimations. We compared the fit of these distributions using the Akaike information criterion (AIC) [21]. Confidence intervals were estimated with 1000 bootstrap resampling from the fitted distribution.

## Results

### Descriptive epidemiology

**Fig. 1.**
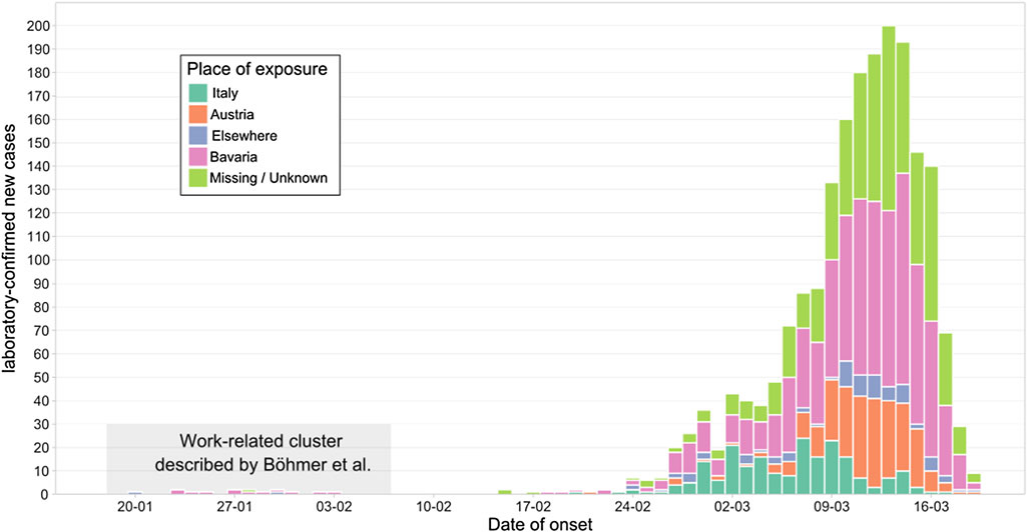
Number of reported COVID-19 cases by date of symptom onset (*n* = 2023) and by place of exposure in Bavaria, Germany, of cases reported between 20 January and 19 March 2020.

In total, 3546 cases were reported from 20 January until 19 March 2020. More than half of the cases were male (54%, *n* = 1904 *vs.* 46%, *n* = 1642). The median age was 46 years (IQR: 30–56) and the mean age was 44 (standard deviation (s.d.): 18.1) years. The date of symptom onset was available in 2023 cases (57%). Figure 1 illustrates the number of cases by date of symptom onset.

The largest proportion of all reported cases (23%) were in the age group from 50 to 60 years and a fifth (21%) were between 40 and 50 years of age. Three percent of reported cases were below the age of 10 (Table 1). Approximately 20% (518/2620) of reported cases were asymptomatic: a larger proportion of asymptomatic cases occurred among those 19 years old and younger (31%), compared with those 20 years of age and older (19%) (Chi-squared test, *P* value <0.01). The proportion of asymptomatic cases among adults under 60 (18%) and over 60 years of age (19%) were not significantly different. The case fatality ratio increased with age, and was highest (31%) among cases 80 years and older.

**Table 1.** Age distribution among COVID-19 cases in Bavaria, Germany, from 20 January to 19 March 2020

	<10	10–19	20–29	30–39	40–49	50–59	60–69	70–79	80–89	> = 90	Missing	Total
	*n* = 99 (3%)	*n* = 247 (7%)	*n* = 562 (16%)	*n* = 555 (16%)	*n* = 713 (21%)	*n* = 798 (23%)	*n* = 294 (8%)	*n* = 168 (5%)	*n* = 92 (3%)	*n* = 9 (0%)	*n* = 9 (0%)	*n* = 3546
Symptom status												
Asymptomatic, *n* (%)	24 (37)	56 (29)	83 (20)	61 (15)	99 (19)	115 (19)	42 (19)	21 (18)	15 (22)	1 (20)	1 (20)	518 (20)
Symptomatic, *n* (%)	41 (63)	135 (71)	328 (80)	337 (85)	427 (81)	497 (81)	179 (81)	97 (82)	53 (78)	4 (80)	4 (80)	2102 (80)
Missing, *n*	34	56	151	157	187	186	73	50	24	4	4	926
Deaths, *n* (%)	0 (0)	1 (0.4)	0 (0)	0 (0)	0 (0)	2 (0.3)	10 (3.4)	14 (8.3)	29 (31.5)	2 (22)	0 (0)	58 (1.6)

**Fig. 2.**
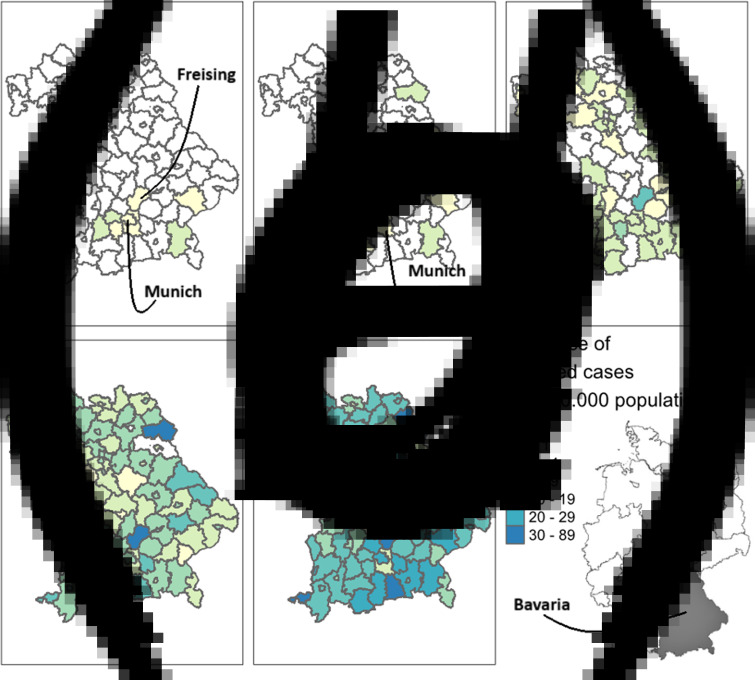
Geographic distribution of COVID-19 cases reported from 20 January to 19 March 2020 in Bavaria, Germany. Map A depicts the cumulative incidence of COVID-19 cases with known date of symptom onset up to 5 February (exclusively showing the first previously described cluster [6]), map B up to 23 February 2020, map C up to 2 March 2020, map D up to 10 March 2020 and map E up to 19 March 2020.

The largest number of cases were reported from Bavaria's capital, the city of Munich (*n* = 850), followed by the surrounding Munich county (*n* = 168) and adjacent Freising county (*n* = 155). Development of the incidence per 100 000 population among cases with known date of symptom onset is illustrated in Figure 2.

Data on travel exposure were available for 1901 (54%) out of 3546 reported cases. Of those, 728 reported travel exposure outside of Bavaria (38%). Most cases had travelled to Austria (*n* = 338), followed by Italy (*n* = 286) and Spain (*n* = 18). Up until 8 March, most cases with exposure abroad had returned from Italy (66%), whereas from 9 March onwards, most cases had returned from Austria (64%, Fig. 1).

### Incubation period and serial interval

**Fig. 3.**
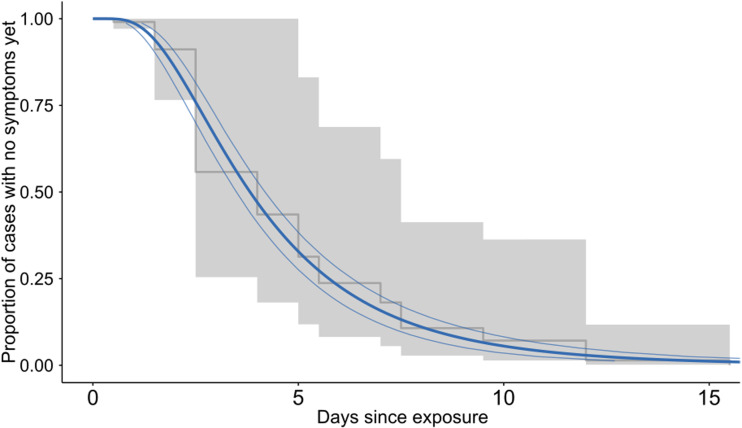
Estimated incubation period of SARS-CoV-2 using the log-normal with 95%CI (blue) parametric distribution and the non-parametric estimate with 95%CI in grey (Kaplan−Meier plot).

We used records of 256 cases with exposure abroad and complete data on travel history, who returned up to 28 days before symptom onset and reported at least one defined symptom, to estimate the incubation period. Among the included cases were individuals who had reported travel to the German federal state North Rhine-Westphalia (*n* = 2), Austria (*n* = 136), Italy (*n* = 106), Spain (*n* = 10) and Iran (*n* = 2) (Sup. Fig. 1). The largest log likelihood was observed when fitting the log-normal distribution. Using the log-normal distribution, the median incubation period of SARS-CoV-2 was 3.8 days (95%CI: 3.5–4.2, Fig. 3). For 95% of infected symptomatic individuals, symptom onset occurred within 10.3 days of exposure (95%CI: 9.1–11.8). The mean of the log-normal distribution was 4.6 days a standard deviation of 3.0 days.

We observed a difference in the incubation period between cases exposed in Italy and Austria (Sup. Fig. 2). Cases related to travels to Italy had a mean incubation period of 5.6 days (s.d.: 3.8) and a median of 4.6 days (95%CI: 4.0–5.3), whereas Bavarian cases who were exposed in Austria had a mean of 3.7 days (s.d.: 2.1) and a median incubation period of 3.2 days (95%CI: 2.9–3.6). Cases who were exposed in Italy had on average a longer duration of stay abroad than those who were exposed in Austria (6 *vs*. 4 days), an earlier date of symptom onset and a different age distribution (Sup. Fig. 3A–C). Twelve per cent of the cases who got infected in Italy were below 20 years of age, while this was 1% of those exposed in Austria and 17% of the cases associated with travels to Austria were between 30 and 40 years of age *vs.* 8% of cases related to travels to Italy (Sup. Fig. 3A–C). We stratified incubation periods by duration of stay abroad, date of symptom onset and age (Sup. Fig. 3D–F). After stratifying by duration of stay abroad, the difference between the estimates for Italy and Austria became smaller for a duration of 4 days and disappeared for a duration of 6 days (Sup. Fig. 4).

**Fig. 4.**
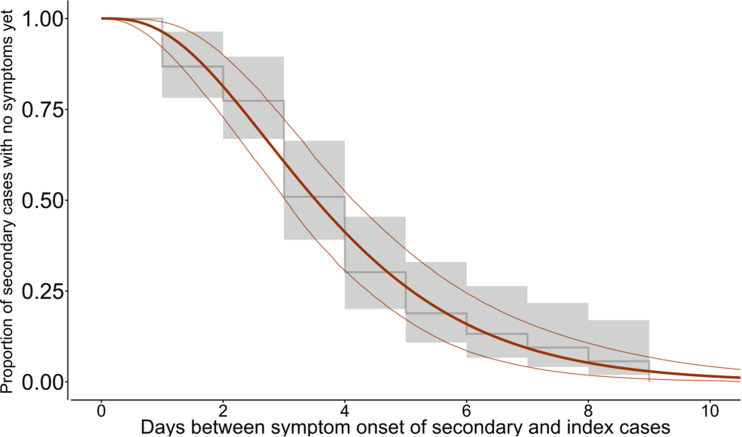
Proportion of secondary cases with no symptom onset yet since symptom onset of the index case among 53 pairs of cases. The grey line depicts the empirical distribution of the serial interval, the orange line the fitted gamma distribution with the 95%CI.

Out of 91 pairs of index and secondary cases, comprising 122 individuals, 53 pairs had sufficient information to estimate the serial interval. Among these pairs, no transmission was observed from children of 10 years and younger (Sup. Fig. 5). The youngest index patient was a 12-year old, who infected two teenagers. The average difference between symptom onset in these 53 pairs was 3.9 days, and ranged from 1 to 9 days. We observed the lowest AIC when fitting the gamma distribution to the data. We estimated that the median serial interval was 3.5 days (95%CI: 3.0–4.1, Fig. 4). In 95% of pairs, the serial interval was less than 8.0 days (95%CI: 6.6–9.4). The mean serial interval was 3.9 days with a standard deviation of 2.2 days.

## Discussion

Exposure to a case from China at the end of January 2020 led to an outbreak of 16 cases in Bavaria that was successfully contained [6]. The testing policy, consisting of testing symptomatic individuals returning from risk areas as well as all contact persons, initially regardless of the presence of symptoms, ensured identification of COVID-19 cases and transmission chains. The first reported COVID-19 cases comprised numerous cases who visited popular travel destinations during a state-wide holiday, and were exposed to community transmission during their travels. These cases drove the initial spread that rapidly led to an exponential increase of COVID-19 cases, reaching 3546 reported cases by 19 March.

Slightly below most estimates of the incubation period [22–26] of around 5 days, we observed a mean incubation period of 4.6 days, and 95% of cases developed symptoms within 11 days. A surprising difference was noted in the incubation periods of cases arising from travel to Italy and Austria. Although some of the difference was explained by the duration of travel, the mean incubation period for cases with exposure in Italy was still higher with the same duration of stay. We may not have been able to identify all families among travellers returning from Italy. Family groups were more common among travellers from Italy, and an unidentified continued exposure to SARS-CoV-2 after a stay abroad would overestimate the incubation period. Travellers from Austria might have been more aware of a possible risk of COVID-19 and thus more attentive to onset of symptoms, as those travellers returned to Bavaria later in the outbreak, and Austria was declared a risk area quicker than some parts of Italy. Finally, a difference in the incubation periods could also be due to differing types of contact or levels of exposure, related to holiday activities in each country [27–29].

Out of 53 pairs of index and secondary cases, we estimated a mean serial interval of 3.9 days. This corresponds with two other studies [30, 31], but is lower than in a systematic review [32]. The serial interval is also dependent on the effectiveness of interventions, such as timing of isolation, and thus, may be changing over time [32, 33]. The mean serial interval is almost one day shorter than the mean overall incubation period (4.6 days) but equal to the mean incubation period for cases coming from Austria (3.7 days). A serial interval shorter than the incubation period suggests that cases may be infectious before the first symptoms are apparent [34]. Our data imply that infectiousness of SARS-CoV-2 peaks around the time of symptom onset.

A strength of our study is the extensive contact tracing by local health authorities, resulting in a high completeness of reporting [12]. This is underlined by the low average age, the high percentage of asymptomatic cases and the low case fatality ratio for reported cases compared with the epidemiological situation in other European countries during the first wave in spring of 2020 [35, 36]. The mean age of 44 was lower than reported from other countries during the early phase of the outbreak [37, 38]. The overall proportion of asymptomatic cases was 20%. This proportion is comparable to a community-based estimate (22%) based on the number of clinical manifestations among sero-positive individuals and a modelling study (18%), where the symptom status was elucidated from COVID-19 cases on board of a cruise ship. The populations under observation in these studies were, on average, older than our study population [39, 40]. The proportion of symptomatic cases was however much higher than estimated by Davies *et al*., who modelled that the proportion of symptomatic infections rose from 21% (credible interval 12–31%) in 10- to 19-year-olds to 69% (credible interval 57–82%) in older adults [41]. And, as underlined by our findings, asymptomatic infections were more present among younger age groups [42].

An inherent caveat of using contact tracing data for the purpose of estimating epidemiological parameters is that many cases were found at an earlier stage, compared with passive surveillance. Asymptomatic infections at the point of sampling may have remained asymptomatic, but some may also have developed symptoms at a later stage that remained unregistered. This selection bias may have shortened our interval estimates and may have increased the proportion of asymptomatic cases in our study.

In both the assessment of the proportion of (a)symptomatic cases and the proportion of cases related to travel, we have chosen to omit cases with a missing value. Some asymptomatic infections may have been classified as cases with missing data on symptom status, as there is no specific data-entry possibility for an asymptomatic infection. However, some symptomatic infections with symptoms other than coughing, diarrhoea, dyspnoea, fever, pneumonia, sore throat or runny nose might have been classified as an asymptomatic case. Besides the potential limitations mentioned, symptomatic persons with a SARS-CoV-2 infection have a higher likelihood of being tested and thus reported. Looking at notification data may therefore overestimate the proportion of symptomatic cases. The proportion of cases with exposure abroad was also subject to cases with missing values. It may be that cases without data on place of exposure were largely exposed in Bavaria (proportion of cases with exposure abroad over all reported cases was 21%). In addition, during the initial outbreak phase testing was focused only on persons with known exposure (contact to a case or travels to risk area) [11]. These two points might have led to an overestimation of the proportion of cases with exposure abroad.

A further caveat of our study concerns the estimation of the incubation period. Cases may have been infected before or after their stay abroad. Some of the cases, especially towards the end of the study period, had a higher chance of being infected prior to their stay abroad than those at the beginning of the study period due to the local rise in cases. However, given the low incidence in late February and early March in Bavaria, Germany [43–45], the high incidence in the visited countries, similar incubation periods among cases stratified by date of symptom onset (Fig. S3E), and the relatively large sample size in the estimation of the incubation period, we expect that an inconsiderable fraction of the cases was infected prior to their stay abroad.

## Conclusion

The analysis of the epidemiology and transmission characteristics of the first COVID-19 cases in Bavaria, Germany, contributes to a further understanding of the novel SARS-CoV-2. In the early weeks of the pandemic, an important proportion of reported cases in Bavaria was attributed to travel outside the region, leading to rapid local transmission. This underlines the importance of continuing efforts in times of low incidence to identify returning travellers who may have been exposed in locations with community transmission, and to quarantine returnees. Duration of quarantine can be deduced from the incubation period. Popular holiday destinations change from winter to summer seasons, so public health authorities should remain abreast of all areas where SARS-CoV-2 infections are continuing or increasing, in order to apply these protection measures and inform the travelling public. These measures are warranted to prevent future numerous importations of SARS-CoV-2, leading to sudden large outbreaks.

## Data Availability

The authors confirm that the data supporting the findings (incubation period, serial interval) of this study are available within the supplementary materials.

## Supplementary material

For supplementary material accompanying this paper visit http://dx.doi.org/10.1017/S0950268821000510. click here to view supplementary material
