# Supplementary material for: Epidemiology and transmission characteristics of early COVID-19 cases, 20 January–19 March 2020, in Bavaria, Germany
Source: Epidemiol Infect. 2021 Mar 2;149:e65. doi: 10.1017/S0950268821000510 (PMC7985897; doi:10.1017/S0950268821000510)

# **Epidemiology and Infection**

Title:

Epidemiology and transmission characteristics of early COVID-19 cases, January 20 – March 19, 2020, in Bavaria, Germany

Authors:

S. Böhm, T. Woudenberg, D. Chen, D. V. Marosevic, M. M. Böhmer, M. Hoch, L. Hansen, J. Wallinga, A. Sing, K. Katz

# **Supplementary Material**

Figure S1. Description of travel history, and date of symptom onset of 256 cases. Every horizontal line depicts one individual. The blue colored area is the duration abroad and the grey area is the time in between return and start of symptoms.


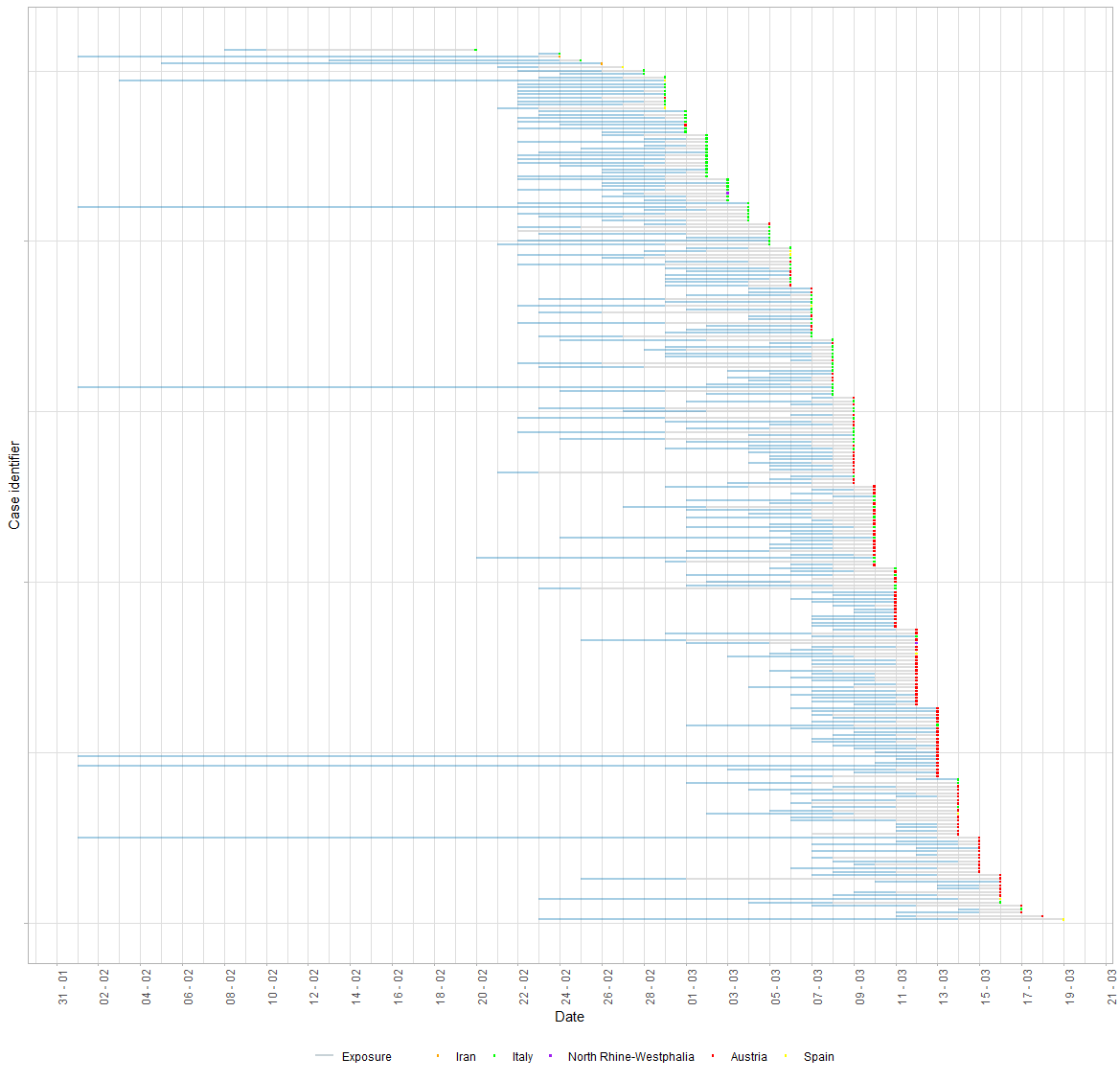


Figure S2. Estimated incubation period of SARS-CoV-2 using the log-normal with 95%CI parametric distribution and the nonparametric estimate (Kaplan Meier plot) stratified by country (Italy in blue and Austria in red).


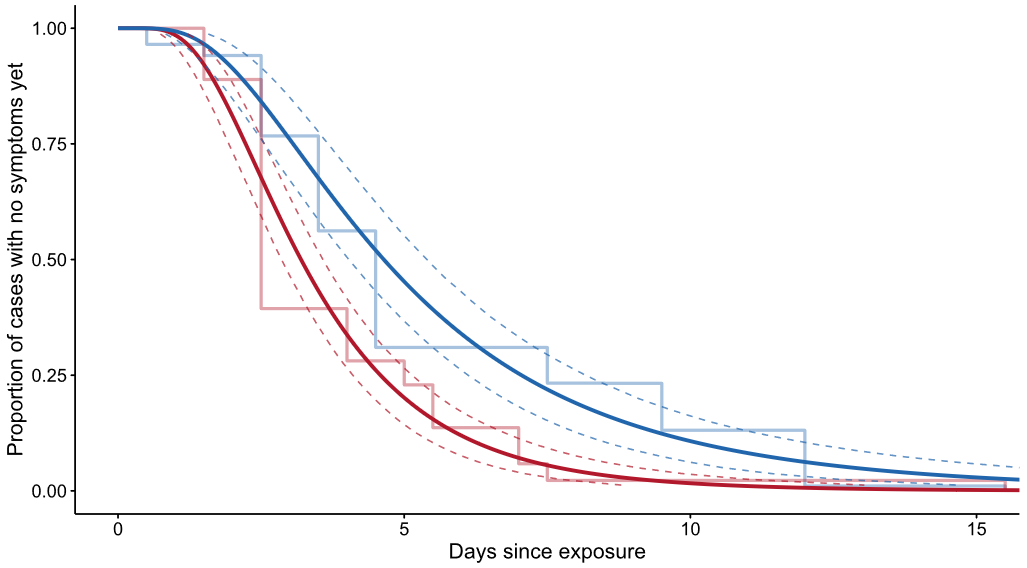


Figure S3. In A-C, the distribution of duration of stay, date of onset, and age groups are shown by country of exposure. In Figures D-F, we visualized the proportion of cases with no symptoms yet since exposure, stratified by duration of stay abroad, date of onset, and age group.


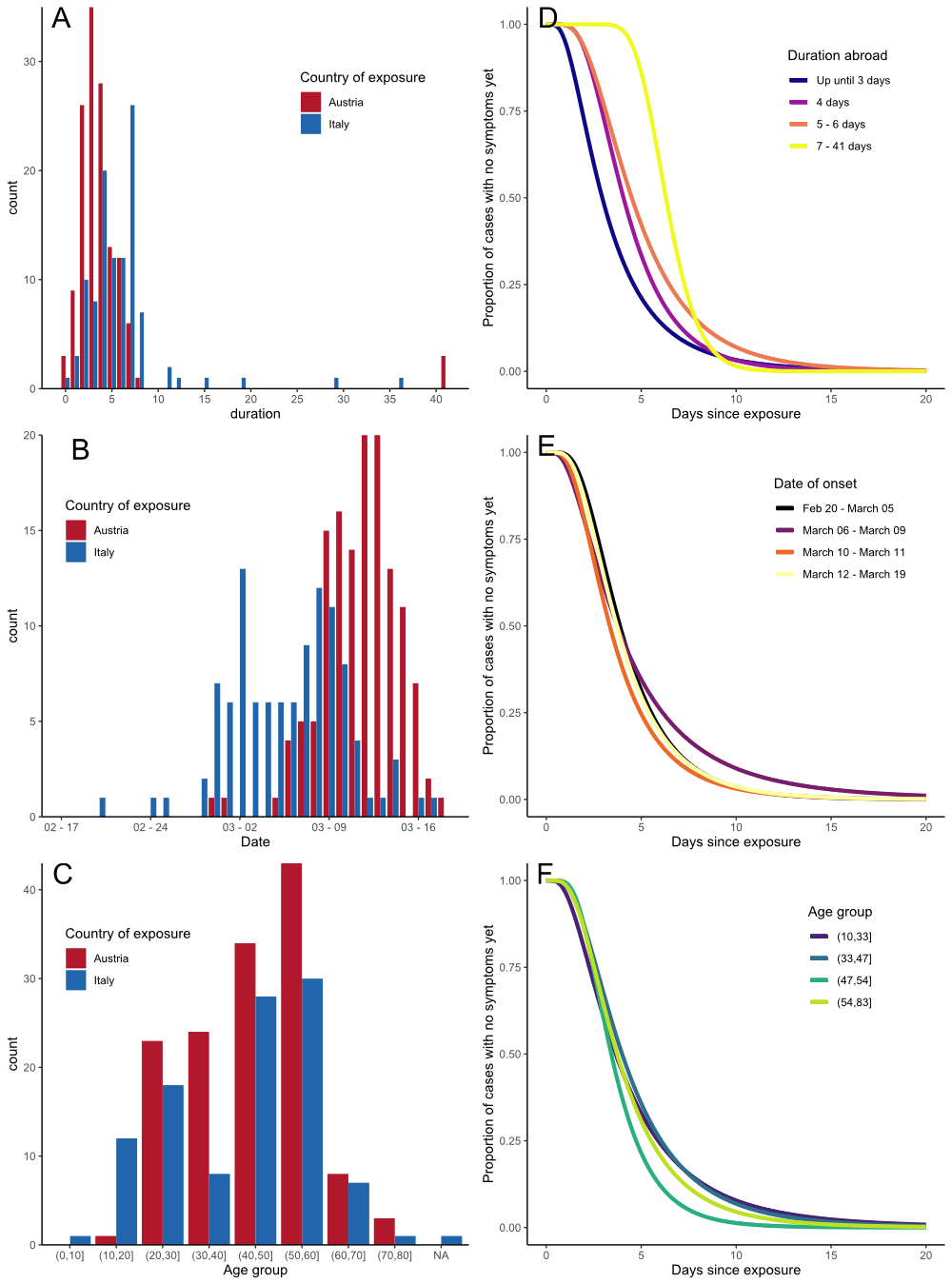


Figure S4. In figure A, the incubation period of cases exposed in Austria (mean = 3.25, n = 70) and Italy (mean = 5.46, n = 21) with a duration of stay of three days or lower are shown. Figure B depicts the incubation period for cases with a duration of stay of 4 days for Austria (mean = 3.8, n = 28) and Italy (mean = 5.8, n = 20). In figure C, the incubation period is shown for cases exposed in Austria (mean = 4.8, n = 25) and Italy (mean = 5.6, n = 24) with a duration of stay of 5-6 days. The stratification by country for cases with a duration of stay of seven and higher had an insufficient number of cases from Austria (n = 10).


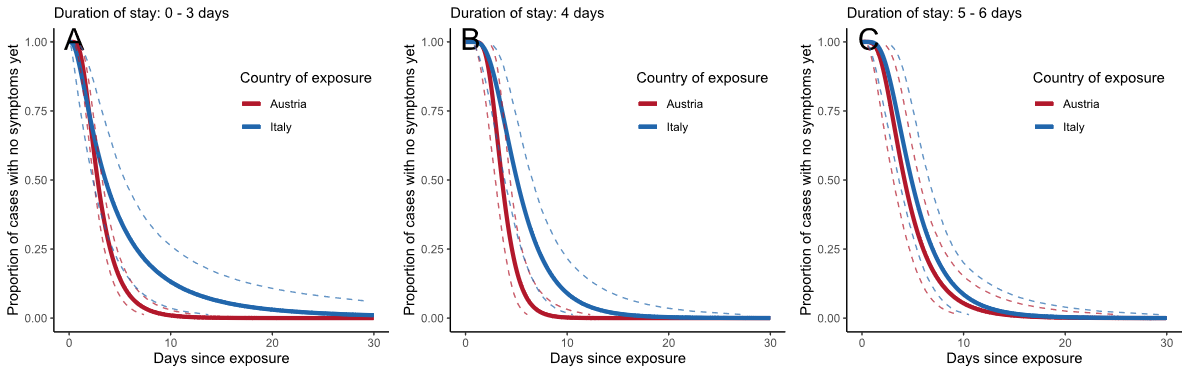


Figure S5. Transmission patterns of COVID-19 cases in Bavaria by age group.


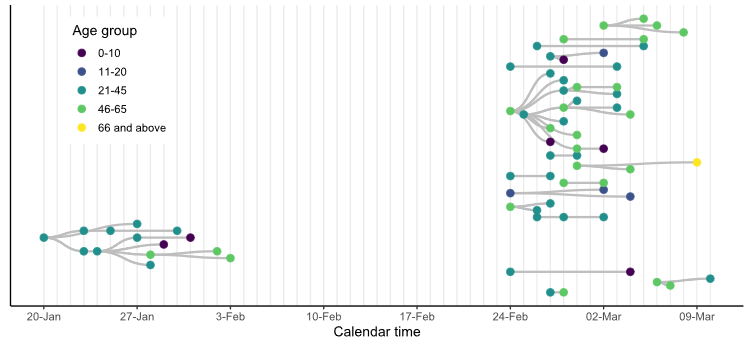

Supplement: Supplementary file 1 [file S0950268821000510sup001.zip › supplement.docx]
